# Performance evaluation of the Q.Clear reconstruction framework versus conventional reconstruction algorithms for quantitative brain PET-MR studies

**DOI:** 10.1186/s40658-021-00386-3

**Published:** 2021-05-07

**Authors:** Daniela Ribeiro, William Hallett, Adriana A. S. Tavares

**Affiliations:** 1 Invicro, Centre for Imaging Sciences, Hammersmith Hospital, London, United Kingdom; 2 Edinburgh Imaging, University of Edinburgh, Edinburgh, UK; 3 University/BHF Centre for Cardiovascular Science, University of Edinburgh, Edinburgh, UK

**Keywords:** PET-MR, Reconstruction, Bayesian, Brain imaging

## Abstract

**Background:**

Q.Clear is a Bayesian penalized likelihood (BPL) reconstruction algorithm that presents improvements in signal-to-noise ratio (SNR) in clinical positron emission tomography (PET) scans. Brain studies in research require a reconstruction that provides a good spatial resolution and accentuates contrast features however, filtered back-projection (FBP) reconstruction is not available on GE SIGNA PET-Magnetic Resonance (PET-MR) and studies have been reconstructed with an ordered subset expectation maximization (OSEM) algorithm. This study aims to propose a strategy to approximate brain PET quantitative outcomes obtained from images reconstructed with Q.Clear versus traditional FBP and OSEM.

**Methods:**

Contrast recovery and background variability were investigated with the National Electrical Manufacturers Association (NEMA) Image Quality (IQ) phantom. Resolution, axial uniformity and SNR were investigated using the Hoffman phantom. Both phantoms were scanned on a Siemens Biograph 6 TruePoint PET-Computed Tomography (CT) and a General Electric SIGNA PET-MR, for FBP, OSEM and Q.Clear. Differences between the metrics obtained with Q.Clear with different *β* values and FBP obtained on the PET-CT were determined.

**Results:**

For in plane and axial resolution, Q.Clear with low *β* values presented the best results, whereas for SNR Q.Clear with higher *β* gave the best results. The uniformity results are greatly impacted by the *β* value, where *β* < 600 can yield worse uniformity results compared with the FBP reconstruction.

**Conclusion:**

This study shows that Q.Clear improves contrast recovery and provides better resolution and SNR, in comparison to OSEM, on the PET-MR. When using low *β* values, Q.Clear can provide similar results to the ones obtained with traditional FBP reconstruction, suggesting it can be used for quantitative brain PET kinetic modelling studies.

**Supplementary Information:**

The online version contains supplementary material available at 10.1186/s40658-021-00386-3.

## Background

Positron emission tomography (PET) is an imaging technique that allows for non-invasive quantitative measurement of biological processes in vivo. Image reconstruction methods can broadly be divided into analytical and iterative algorithms. Whereas analytical reconstruction algorithms (e.g. filtered back-projection, FBP) assume continuous data and introduce a discrete character to it a posteriori, iterative reconstruction algorithms (e.g. ordered subset expectation maximization, OSEM) assume discretely sampled data. Although iterative reconstruction algorithms are routinely used in the clinical setting, where image quality and lesion contrast are of great importance, analytical reconstruction algorithms are still used in research for accurate PET data quantification via kinetic modelling [1].

The block sequential regularized expectation maximization (BSREM) algorithm is a Bayesian penalized likelihood (BPL) method that uses prior knowledge as a relative difference penalty term in the cost function, weighted by a penalization parameter *β* [2]. Unlike expectation-maximization (EM) algorithms that typically become noisy as the number of iterations is increased, the penalty term suppresses noise allowing the BSREM algorithm to iterate to convergence, in principle increasing the accuracy of the quantitative image measurements [2, 3]. Although BPL algorithms are not new, their use in clinical and research settings has been limited due to the computational cost involved and lack of availability in clinical systems [2]. Recently, General Electric (GE) Healthcare has released the BSREM penalized likelihood reconstruction algorithm under the product name of Q.Clear. However, due to its recent release, its impact in clinical use and research applications is still being evaluated [2]. The FBP reconstruction is not available for clinical use on the GE SIGNA PET-MR scanner; hence, OSEM reconstructions have been used for processing brain studies. In smaller regions, such as the ones that can be found in the brain, the convergence rate of OSEM process must be stopped early in order to not compromise image quality due to excessive noise [4, 5]. Although OSEM is being used for processing of both whole-body and brain scans, studies such as the ones conducted by Reilhac et al. [6] and Walker et al. [7] have reported a positive bias in regions with low activity and a negative bias in regions of high activity in low-count scans which had been reconstructed with this algorithm. Jian et al. [8] however found a negative bias in both high-count and low-count regions, in scans which had been acquired and reconstructed under a similar paradigm as described above [6–8]. This is of particular importance with radiotracers which are mass dependent due to the potential of pharmacological effects. The restricted injected dose limits may therefore result in noisy imaging data with low count statistics. Despite multiple advances in iterative methods of quantification (e.g. OSEM and BPL), FBP is still used as method of choice for accurate brain PET kinetic modelling studies due to its linear response. The impact of using non-FBP methods for reconstruction of quantitative brain studies is poorly understood and with latest PET-MR technology rapidly gaining momentum in the field of brain clinical research, studies are needed to assess and minimise the gap between traditional PET-CT kinetic modelling studies with data reconstructed using FBP versus PET-MRI OSEM and Q.Clear approaches.

Furthermore, brain PET imaging plays a critical role in clinical diagnosis of dementia and other neurological disorders. Despite that, to date, studies looking to assess Q.Clear performance in clinical PET have been primarily focused on whole-body analysis and fluorinated radiotracers [9–13]; therefore, there is a need to assess the performance of this framework in the context of neuroimaging and with different PET isotopes. This study aimed to evaluate the performance of the Q.Clear, against that of the widely used OSEM and the FBP algorithms in brain phantom images acquired on a clinical PET-CT and on a clinical PET-MR system using ^18^F- and ^11^C-labelled radiotracers. We hypothesise that despite differences in scanner design and performance as well as reconstruction frameworks, brain PET quantitative outcomes can be approximated by assessing the performance of different reconstruction algorithms and identifying those that result in least impact on successful quantitative PET-MR brain studies.

## Materials and methods

The PET-CT and PET-MR data reported here was collected at a single site. The primary source for radiation measurements performed in the department is with a ^137^Cs source that is used for the daily quality control procedures on the dose calibrators. The nominal activity of this source was previously adjusted as part of a cross calibration exercise, to the secondary standard ionisation chamber at the National Physical Laboratory, in the UK. The remaining measurement equipment including the PET-CT and PET-MR scanners are then calibrated using measurements made from the dose calibrator and a cylindrical phantom filled with ^18^F or ^11^C tracer. Additionally, and for the purposes of this single-centre study, a large phantom volume-of-interest (VOI) for ^18^F and ^11^C was used, prior to starting the reconstruction comparison [14].

### PET-CT and PET-MR phantom data acquisition and reconstruction

The National Electrical Manufacturers Association (NEMA) Image Quality (IQ) phantom was prepared by adding [^18^F]BCPP-EF (49.5 ± 5.4 MBq, mean ± SD, *n* = 2) solution to the phantom, ensuring that the hot spheres contained a concentration four times that of the background (22.4 kBq/mL *versus* 5.6 kBq/mL) [15]. The two larger spheres were filled with non-radioactive water, henceforth referred to as cold spheres. This phantom was scanned for 40 min once in the department single-centre benchmark PET-CT scanner (Siemens 6 Biograph TruePoint, Siemens Healthcare, Germany; detector size 4.0 × 4.0 × 20 mm^3^ (transverse, axial, depth directions) and NEMA NU 2–2007 full-width half maximum at 1 cm from centre of 4.1 mm transverse and 4.7 mm axial [16]) and once in the department single-centre benchmark PET-MR scanner (GE SIGNA, GE Healthcare, USA; detector size 4.0 × 5.3 × 25 mm^3^ and NEMA NU 2–2007 full-width half maximum at 1 cm from centre of 4.05 mm transverse and 6.08 mm axial [17]). In both scanners the data was acquired in listmode and a matrix of 128 × 128 was used for reconstruction.

The Hoffman phantom was prepared by mixing 29.6 MBq of [^18^F]BCPP-EF, or 34.4 MBq of [^11^C]SA4503, or 36.4 MBq of [^11^C]UCB-J in water and then filling the phantom, ensuring the removal of large air bubbles. The ^18^F phantom was scanned for 40 min in the PET-CT scanner, reconstructed with a matrix of 256 × 256 and for 40 min in the PET-MR scanner, reconstructed with a matrix of 384 × 384, in order to keep the voxel size as similar as possible across all PET datasets. The matrix size on *z*-direction for Hoffman scans acquired in the PET-MR is 89, for Hoffman scans acquired in the PET-CT is 109, for NEMA IQ acquired in the PET-MR is 89 and for NEMA IQ acquired in the PET-CT is 111. The voxel size for the Hoffman scans acquired in the PET-MR is 1 × 1 × 2.78 mm^3^, for the Hoffman scans acquired in the PET-CT is 1.02 × 1.02 × 2.03 mm^3^, for the NEMA IQ acquired in PET-MR is 4.69 × 4.69 × 2.78 mm^3^ and for the NEMA IQ acquired in the PET-CT is 5.35 × 5.35 × 5 mm^3^. Due to the short half-life of ^11^C, the Hoffman phantom was filled with [^11^C]SA4503 solution and scanned in the PET-MR and subsequently filled with [^11^C]UCB-J solution and scanned in the PET-CT. The duration of the acquisition and acquisition parameters were the same as for the ^18^F phantom and the data was acquired in listmode for both the ^11^C and ^18^F phantoms.

Each NEMA and Hoffman phantom scans acquired on the PET-CT scanner was reconstructed 6 times and each NEMA and Hoffman phantoms acquired on the PET-MR scanner was reconstructed 13 times, as can be observed in Table 1. The FBP reconstructions were only performed on the PET-CT scanner and the time of flight (TOF with time resolution of < 386 ps) Q.Clear reconstructions were only performed on the PET-MR. The three-dimensional (3D) OSEM reconstructions were performed on the PET-CT and TOF-OSEM reconstructions were performed on the PET-MR. OSEM with 4 iterations and 16 subsets was selected based on previously reported data comparing TOF and non-TOF measurements in different PET systems [5, 18–20]. Furthermore, the Q.Clear algorithm has been devised to improve image quality, without increasing noise, by using a penalty function. This penalty function behaves as a noise suppression term. To estimate correspondence of Q.Clear *β* value (up to 1000) and the size of the FBP and OSEM filter kernel for two different isotopes and brain phantoms in a variety of outcome measures (e.g. resolution, noise and uniformity), a wide range of filter from 5 to 15 mm was used in this study. Attenuation correction on the PET-CT was performed with a low-dose attenuation correction CT scan performed prior to the PET acquisition (NEMA phantom: 30 mAs, 130 kV, 5 mm slice, 1.5 pitch and 1.5 s rotation time; Hoffman phantom: 30 mAs, 130 kV, 3 mm slice, 0.55 pitch and 0.8 s rotation time). Attenuation correction on the PET-MR was performed with a GE CT-based template of the respective phantoms. All images acquired in the PET-CT and in the PET-MR have been reconstructed with random and scatter correction. These protocols were designed based on centre benchmark during this single-centre project and based on previous literature as detailed above.

**Table 1:** Summary of methods used for reconstructing the NEMA and Hoffman phantom datasets

Reconstruction method	Nomenclature	^**18**^F NEMA PET-CT	^**18**^F NEMA PET-MR	^**18**^F Hoffman PET-CT	^**18**^F Hoffman PET-MR	^**11**^C Hoffman PET-CT	^**11**^C Hoffman PET-MR
FBP with 5 mm filter (PET-CT)	FBP_5mm	x		x		x	
FBP with 10 mm filter (PET-CT)	FBP_10mm	x		x		x	
FBP with 15 mm filter (PET-CT)	FBP_15mm	x		x		x	
3D OSEM 4 iterations, 8 subsets, 5 mm filter (PET-CT)	OSEM_4i8s5mm	x					
3D OSEM 4 iterations, 8 subsets, 10 mm filter (PET-CT)	OSEM_4i8s10mm	x					
3D OSEM 4 iterations, 8 subsets, 15 mm filter (PET-CT)	OSEM_4i8s15mm	x					
3D OSEM 4 iterations, 16 subsets, 5 mm filter (PET-CT)	OSEM_4i16s5mm			x		x	
3D OSEM 4 iterations, 16 subsets, 10 mm filter (PET-CT)	OSEM_4i16s10mm			x		x	
3D OSEM 4 iterations, 16 subsets, 15 mm filter (PET-CT)	OSEM_4i16s15mm			x		x	
ToF 3D OSEM 4 iterations, 8 subsets, 5 mm filter (PET-MR)	OSEM_4i8s5mm		x				
ToF 3D OSEM 4iterations, 8 subsets, 10 mm filter (PET-MR)	OSEM_4i8s10mm		x				
ToF 3D OSEM 4 iterations, 8 subsets, 15 mm filter (PET-MR)	OSEM_4i8s15mm		x				
ToF 3D OSEM 4 iterations, 16 subsets, 5 mm filter (PET-MR)	OSEM_4i16s5mm				x		x
ToF 3D OSEM 4 iterations, 16 subsets, 10 mm filter (PET-MR)	OSEM_4i16s10mm				x		x
ToF 3D OSEM 4 iterations, 16 subsets, 15 mm filter (PET-MR)	OSEM_4i16s15mm				x		x
ToF 3D Q.Clear with β100 (PET-MR)	QClear100		x		x		x
ToF 3D Q.Clear with β200 (PET-MR)	QClear200		x		x		x
ToF 3D Q.Clear with β300 (PET-MR)	QClear300		x		x		x
ToF 3D Q.Clear with β400 (PET-MR)	QClear400		x		x		x
ToF 3D Q.Clear with β500 (PET-MR)	QClear500		x		x		x
ToF 3D Q.Clear with β600 (PET-MR)	QClear600		x		x		x
ToF 3D Q.Clear with β700 (PET-MR)	QClear700		x		x		x
ToF 3D Q.Clear with β800 (PET-MR)	QClear800		x		x		x
ToF 3D Q.Clear with β900 (PET-MR)	QClear900		x		x		x
ToF 3D Q.Clear with β1000 (PET-MR)	QClear1000		x		x		x

### Data analysis

The NEMA phantom scans were analysed using a customised Interactive Data Language (IDL ®) program according to NEMA standards [21, 22]. Circular regions of interest (ROIs), equal in diameter to each sphere, and 60 adjacent background ROIs were drawn. Contrast and background variability were calculated using the NEMA NU 2-2012 equations [21].

The percentage contrast for each hot sphere was calculated according to Eq. 1:

1 $$ \% contrast\ for\  hot\  sphere=\frac{\frac{C_H}{C_B}-1}{\frac{a_H}{a_B}-1}\times 100 $$

where *C*_*H*_ is the average of the counts found in the ROI for a hot sphere, *C*_*B*_ is the average of the background counts in the background ROI for the same sphere, *a*_*H*_ and *a*_*B*_ the activity concentration in the hot sphere and in the background, respectively [21].

The Percentage contrast for each cold sphere was calculated according to Eq. 2:

2 $$ \% contrast\ for\ cold\ sphere=\left(1-\frac{C_C}{C_B}\right)\times 100 $$

where *C*_*C*_ represents the average of counts in the ROI for a cold sphere and *C*_*B*_ represents the average of the 60 background ROI counts for the same sphere size [21].

For the background variability, the standard deviation of the background ROI counts for each sphere size was calculated according to Eq. 3,

3 $$ SD=\sqrt{\sum_{k=1}^K\frac{\left({C}_{B,k}-{C}_B\right)2}{K-1}} $$

where *k* equals the 60 background ROI counts and the background variability was calculated according to Eq. 4:

4 $$ \% background\ variability=\frac{\mathrm{SD}}{C_B}\times 100 $$

The Hoffman phantom data were analysed using the VivoQuant® software version 3.5 patch 2 (inviCRO LLC, USA) [23]. The resolution (expressed as full-width-half-maximum, FWHM) was determined by correlation of the acquired images with a digital version of the Hoffman phantom convolved with different Gaussian filters. This allowed for comparing estimated in-plane and axial resolutions (VivoQuant Hoffman Phantom Analysis Workflow Working Instruction Document, unpublished). The axial uniformity metric was determined by drawing a VOI in the right putamen (size of 2400 mm^3^) and calculating the percentage standard deviation according to Eq. 5 [24]:

5 $$ \% standard\ deviation=\frac{\sigma_p\ }{C_P}\times 100 $$

Where *C*_*P*_ is the average counts in the VOI and *σ*_*p*_ the standard deviation.

The signal-to-noise ratio (SNR) was determined by drawing a VOI in the right putamen and a VOI in the background “white matter” region of the Hoffman phantom (devoid of radioactivity) and it was calculated according to Eq. 6:

6 $$ SNR=\frac{C_P-{C}_W}{\sigma_W} $$

Where *C*_*P*_ is the average counts in the VOI for the putamen, *C*_*W*_ is the average counts in the VOI placed in a uniform area in the background and *σ*_*W*_ the standard deviation in the background [25].

Differences in contrast, background variability, resolution, uniformity and SNR were calculated relative to the FBP reconstruction with 5 mm FWHM Gaussian filter, the standard FBP reconstruction for the department. Bland-Altman plots were used to investigate the quantitative differences between the FBP with 5 mm FWHM Gaussian filter (obtained in the PET-CT) and the TOF-OSEM with 4 iterations, 8 subsets and 5 mm filter (obtained in the PET-MR) versus Q.Clear with different *β* values.

GraphPad Prism version 8.1.0 for Windows (GraphPad Software, USA) was used for statistical analysis and graphical representation [26].

## Results

### NEMA and Hoffman phantom results with ^18^F-solution

**Fig. 1:**
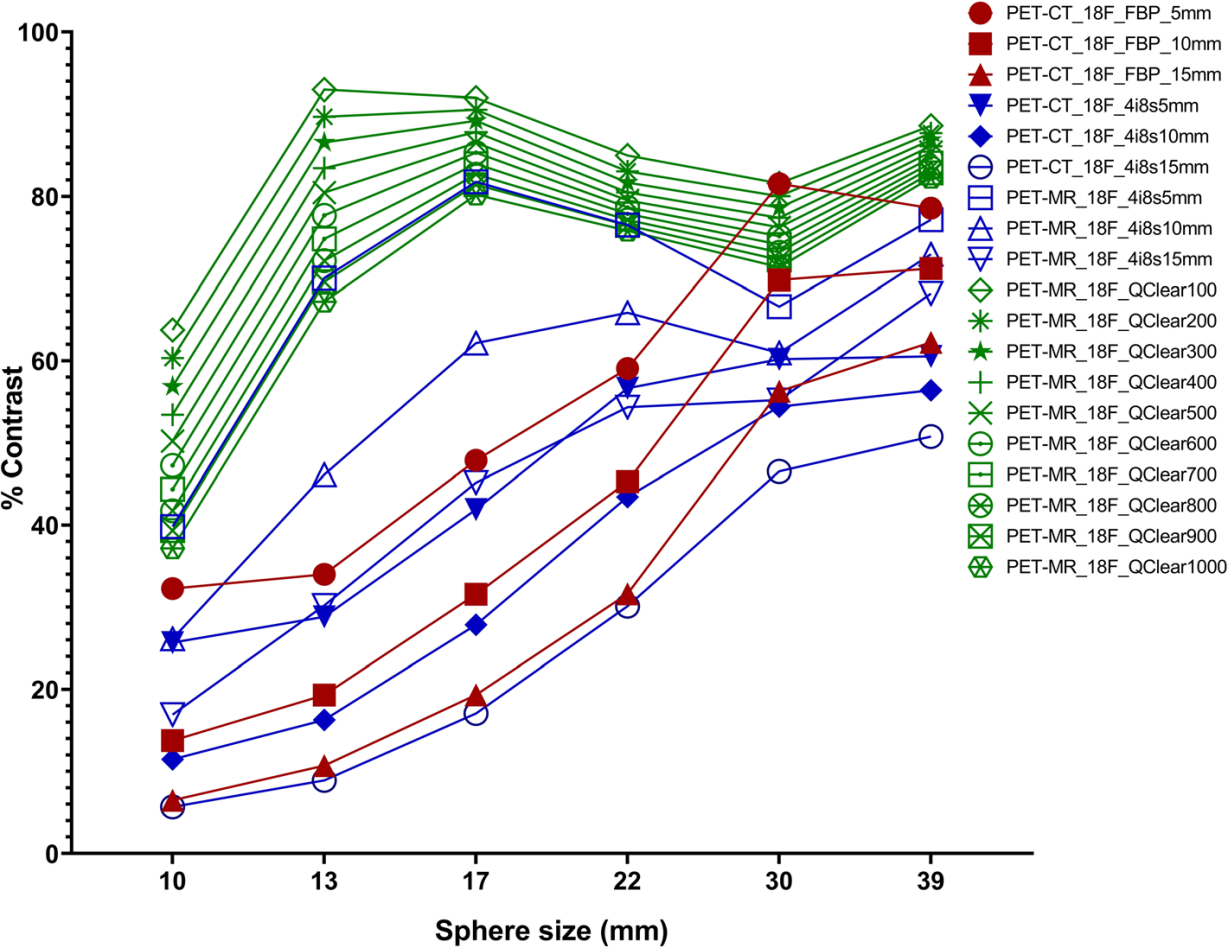
NEMA phantom measured percentage contrast recovery for all reconstruction methods when using ^18^F-solution. Note highest percentage contrast of Q.Clear methods compared with OSEM and FBP methods

The Q.Clear reconstructions (varying *β* values) from the PET-MR provided consistently higher percentage contrast compared to OSEM reconstructions on the PET-CT and the PET-MR, as well as the FBP on the PET-CT. For all reconstruction methods, the percentage contrast was highest for large diameter spheres of the NEMA phantom and reduced with sphere size (Fig. 1). The largest variability in the percentage contrast across all reconstruction methods was measured for the 13 mm sphere (mean 55.7%, standard deviation 29.4%, median 69.6% and coefficient of variation 52.8%) compared to the smallest variability for the 30 mm sphere (mean 69.0%, standard deviation 10.5%, median 72.3% and coefficient of variation 15.2%). The lowest quantitative differences were found for Q.Clear with β1000 when comparing with FBP with a 5 mm kernel and TOF-OSEM with 4 iterations, 8 subsets and 5 mm kernel (13.5 and 0.36, respectively) (Supplementary Files 1 and 2).

**Fig. 2:**
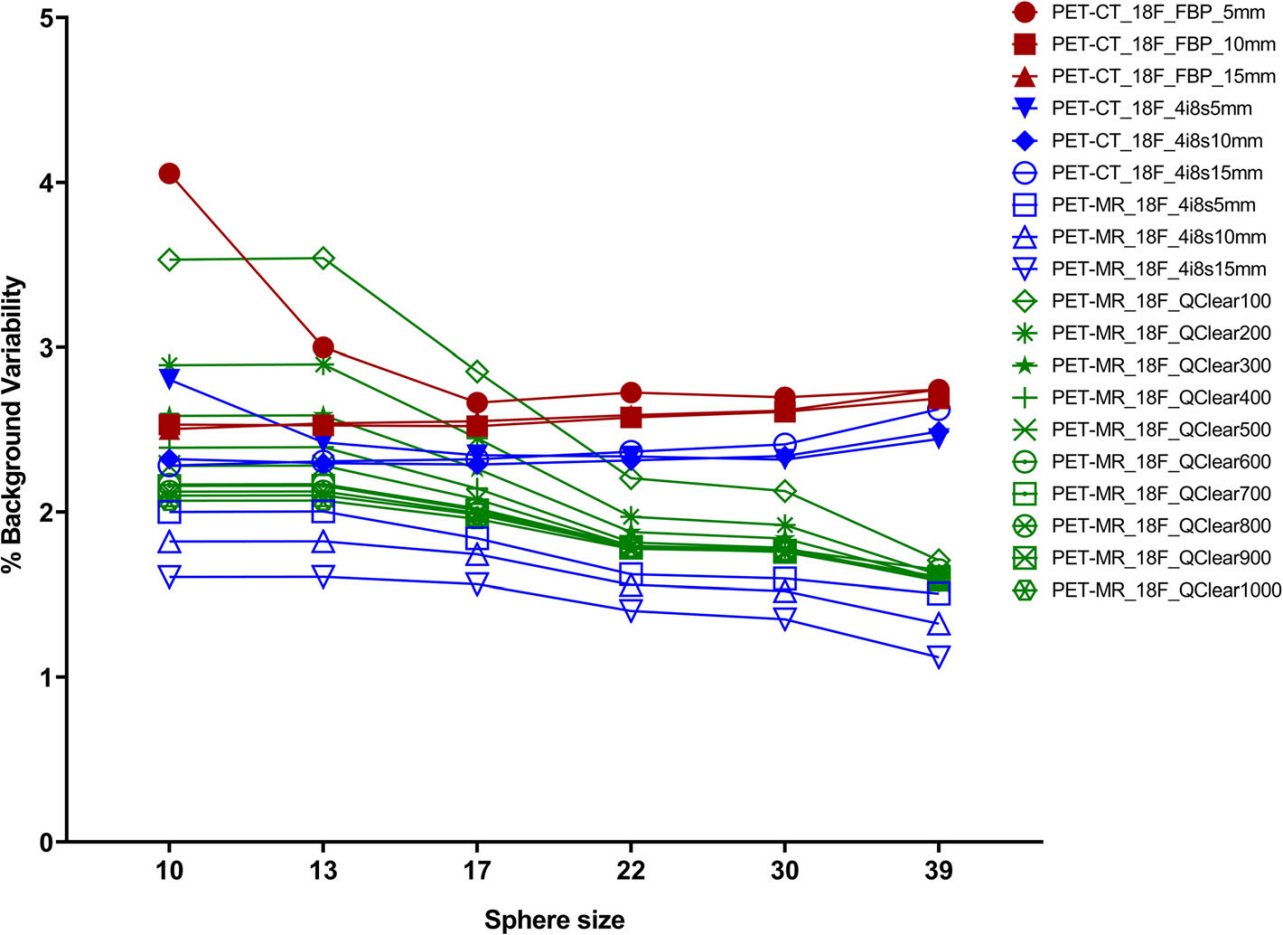
NEMA phantom measured background variability for all reconstruction methods when using ^18^F-solution. Note OSEM reconstructions performed on the PET-MR scanner resulted in the lowest background variability of all methods

Analysis of the NEMA phantom background showed the OSEM on the PET-MR resulted in the smallest background variability of all methods (Fig. 2). The largest background variability was measured for FBP with the smallest filter kernel, followed by the Q.Clear method with the lowest *β* value of 100. For each sphere size, the measured mean background variability dropped from 2.43 % (10 mm sphere) to 1.89% (39 mm sphere). The same trend was observed for the standard deviation (0.58 to 0.53 %) and median (2.28 to 1.61%), while the coefficients of variation were relatively stable at 23.8%, 18.6%, 15.1%, 19.0%, 20.1% and 28.4% for the 10, 13, 17, 22, 30, and 39 mm sphere, respectively. The lowest quantitative difference was found for Q.Clear with β100 (0.32) when comparing with FBP with a 5-mm kernel and for Q.Clear with β1000 (0.11) when TOF-OSEM with 4 iterations, 8 subsets and 5 mm kernel (Supplementary Files 3 and 4).

**Fig. 3:**
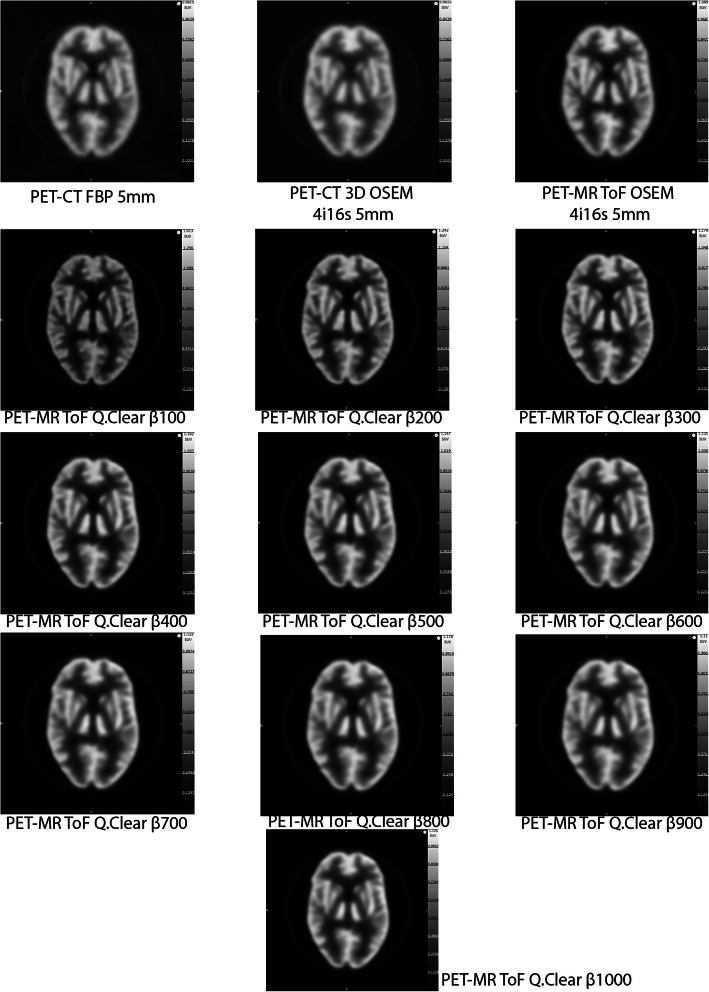
Hoffman phantom filled with ^18^F-BCPP in the PET-CT and PET-MR. FBP and 3D OSEM 4 iterations, 16 subsets, 5 mm filter obtained in the PET-CT are displayed. TOF OSEM 4 iterations, 16 subsets, 5 mm filter and TOF Q.Clear β100 to 1000 obtained in the PET-MR are also displayed. Note the visual differences in image quality for the Q.Clear reconstructions as β increases

**Fig. 4:**
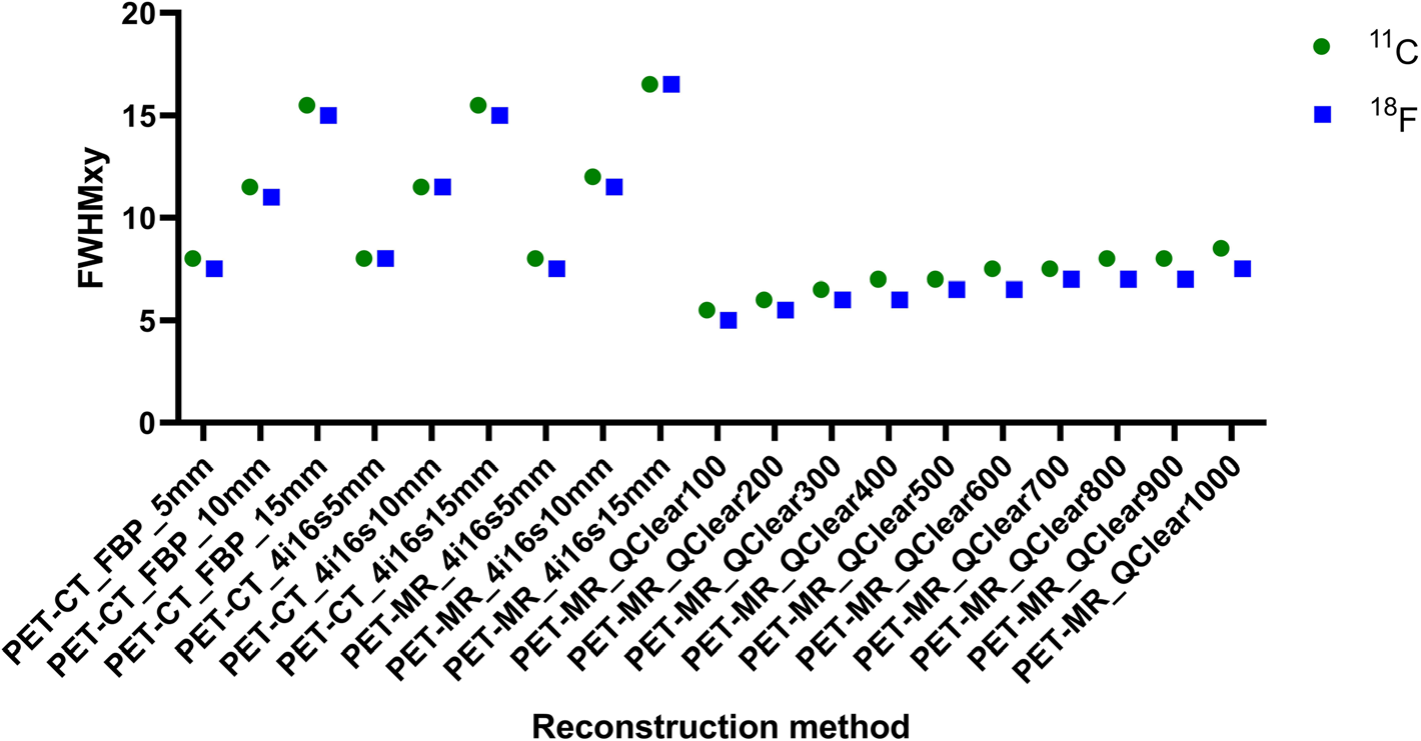
Hoffman phantom measured FWHM (*x*,*y*) for all reconstruction methods when using a ^*11*^C and a ^*18*^F-solution. Note the best resolution was obtained with the Q.Clear with β100, for both radionuclides

Images of the Hoffman phantom filled with the ^18^F solution and reconstructed with different methods are presented in Fig. 3. The highest FWHM (*x*,*y*) (worst transaxial spatial resolution) of 16.5 mm observed for the Hoffman phantom, was with ^18^F in the PET-MR, for OSEM 4 iterations, 16 subsets and a 15 mm filter (Fig. 4). The lowest FWHM of 5 mm was for Q.Clear with *β* of 100. A FWHM of 7.5 mm was measured for FBP with 5 mm filter. Relative to the FBP with 5 mm filter reconstruction, the largest difference (− 9.0 mm) was for PET-MR OSEM 4 iterations, 16 subsets and 15 mm filter; while the smallest difference (0.0 mm) was for PET-MR OSEM 4 iterations, 16 subsets and 5 mm filter together with Q.Clear *β* value of 1000.

**Fig. 5:**
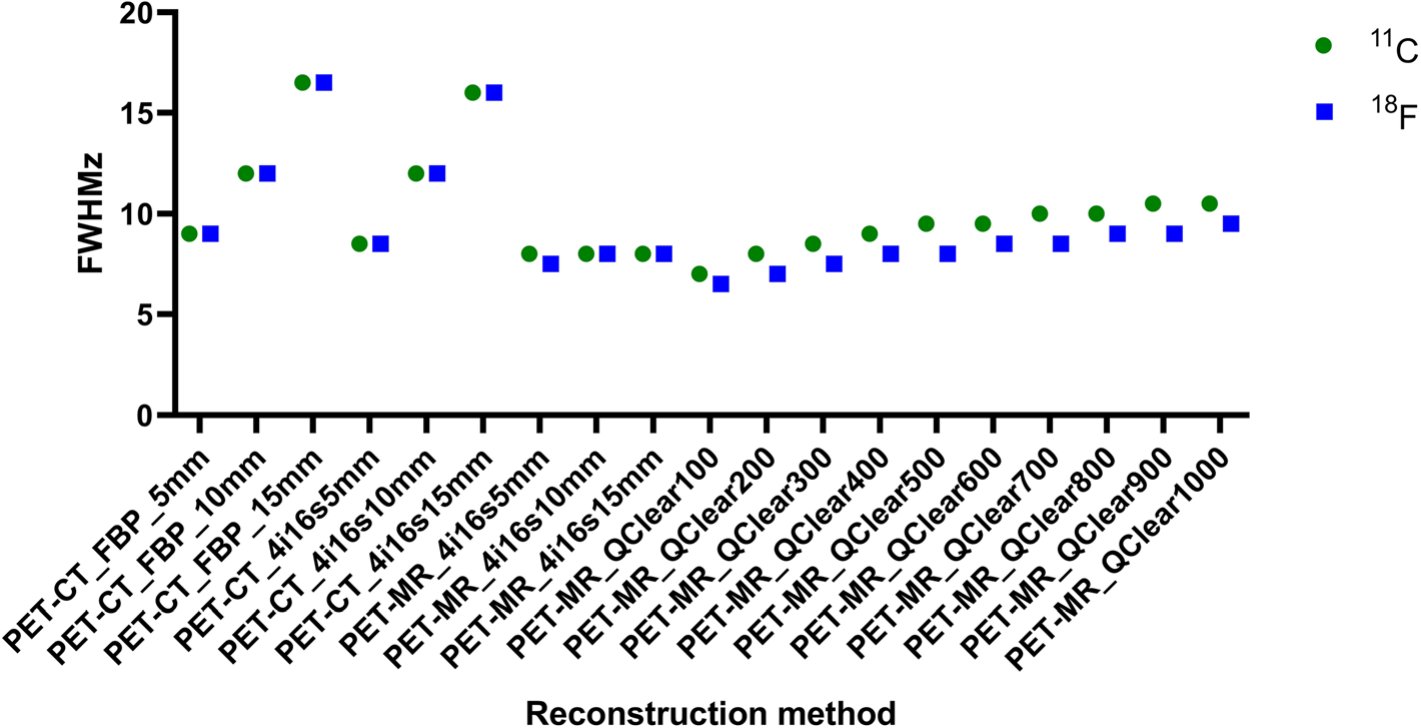
Hoffman phantom measured FWHM (*z*) for all reconstruction methods when using a ^*11*^C and a ^*18*^F-solution. Note the best resolution was obtained with the Q.Clear with β100, for both radionuclides

The highest FWHM (*z*) (worst *z*-axis spatial resolution) of 16.5 mm was observed for FBP with a 15-mm filter (Fig. 5). The lowest FWHM (*z*) of 6.5 mm was for Q.Clear reconstruction with *β* of 100. Relative to FBP with a 5-mm filter, the largest difference was for FBP with a 15-mm filter (− 7.5 mm), while the smallest was for the Q.Clear with β800 or 900 (0.0 mm).

**Fig. 6:**
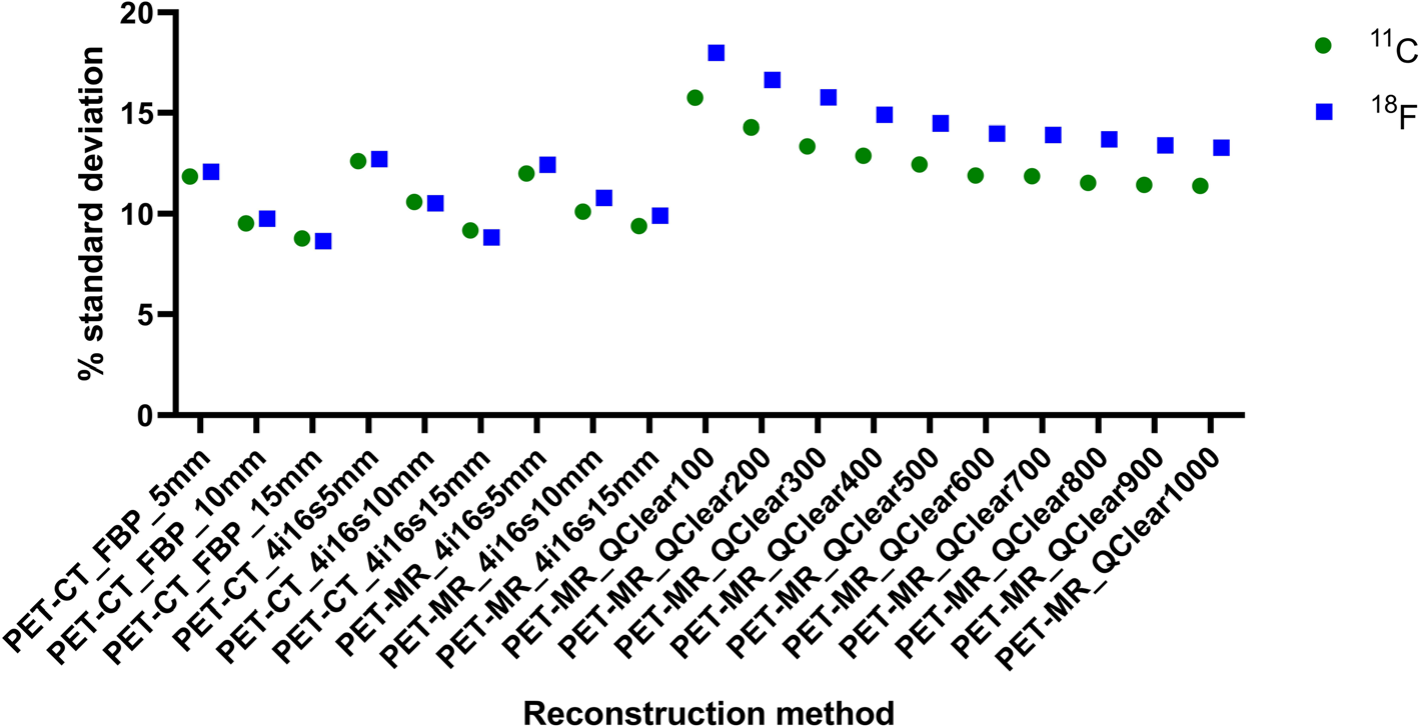
Hoffman phantom measured uniformity for all reconstruction methods when using a ^11^C and a ^18^F-solution. Note the best uniformity was obtained with FBP with a 15-mm filter

The Q.Clear with *β* of 100 yielded the poorest uniformity of 18.0%, while the best uniformity was measured for FBP with a 15-mm filter (8.6%) (Fig. 6). Relative to FBP with 5 mm filter, the largest difference (− 5.9) was for PET-MR Q.Clear with *β* of 100, while the smallest difference (− 0.3) was for PET-MR OSEM 4 iterations, 16 subsets, 5 mm filter.

**Fig. 7:**
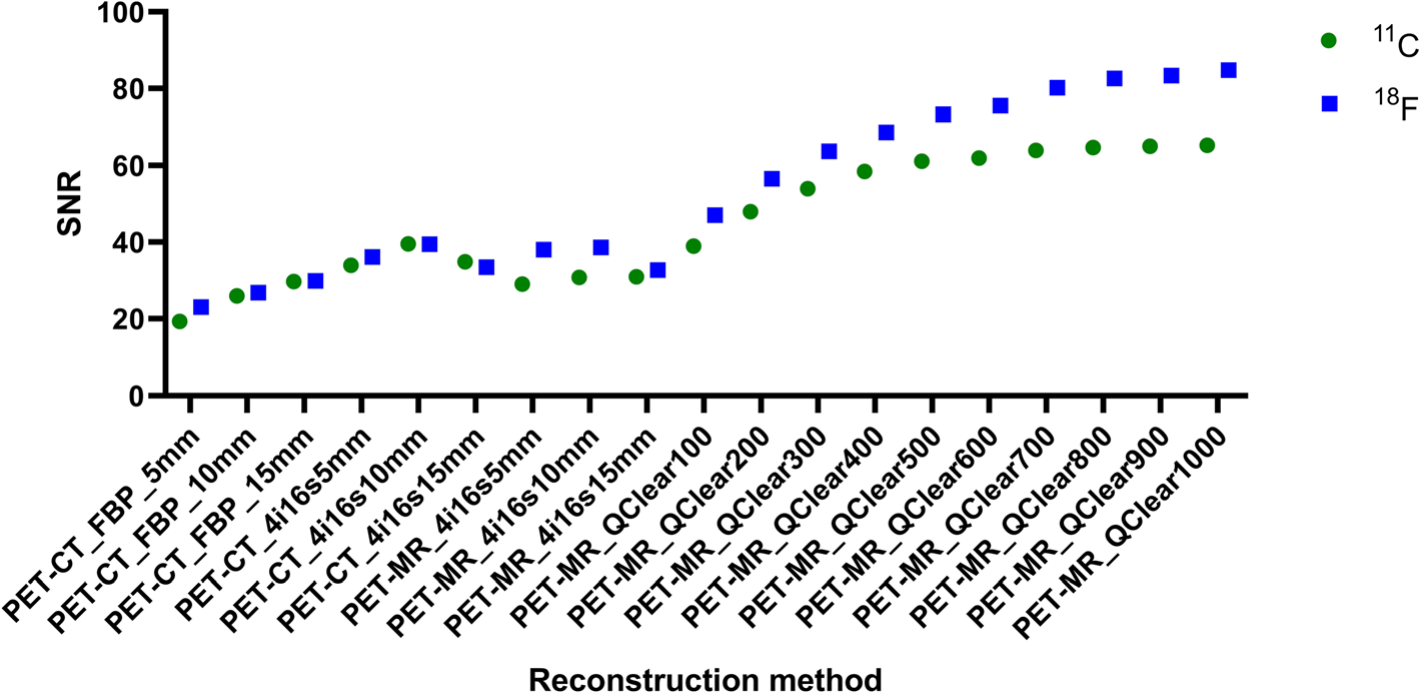
Hoffman phantom measured signal-to-noise for all reconstruction methods when using a ^11^C and a ^18^F-solution. Note the best signal-to-noise was obtained with Q.Clear with β1000

For SNR, the largest value (84.8) was for Q.Clear with *β* of 1000 (Fig. 7). The poorest SNR was for FBP with 5 mm filter (23.0). Relative to FBP with 5 mm filter, the largest difference (− 61.8) was for Q.Clear with *β* of 1000, while the smallest difference (− 3.8) was for FBP with 10 mm filter.

### Hoffman phantom results with ^11^C-solution

**Fig. 8:**
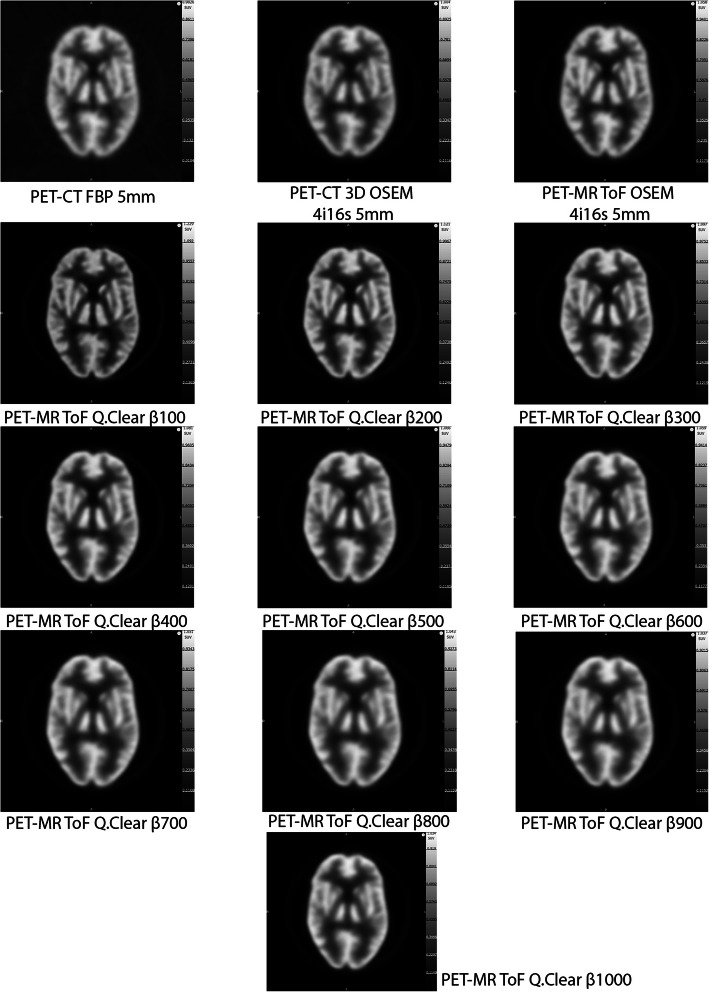
Hoffman phantom filled with ^11^C-SA4503 and ^11^C-UCB-J in the PET-CT and PET-MR. FBP and 3D OSEM 4 iterations, 16 subsets, 5 mm filter obtained in the PET-CT are displayed. TOF OSEM 4iterations 16 subsets, 5 mm filter, and TOF Q.Clear β100 to 1000 obtained in the PET-MR are also displayed. Note the visual differences in image quality for the Q.Clear reconstructions as β increases

Images of the Hoffman phantom filled with the ^11^C solutions and reconstructed with different methods are presented in Fig. 8. The highest FWHM (*x*,*y*) was 16.5 mm for PET-MR OSEM reconstruction with 4 iterations, 16 subsets and 15 mm filter (Fig. 4). The lowest FWHM of 5.5 mm was for Q.Clear with *β* of 100. A FWHM of 8 mm was measured for FBP with 5 mm filter. Relative to this, the largest difference (− 8.5 mm) was for PET-MR OSEM 4 iterations, 16 subsets and 15 mm filter, while the smallest difference (0.0 mm) was for PET-CT OSEM 4 iterations, 16 subsets and 5 mm filter together with PET-MR OSEM 4 iterations, 16 subsets and 5 mm filter and Q.Clear with β800 and 900.

The highest FWHM (*z*) was 16.5 mm for FBP and 15 mm filter (Fig. 5). The lowest FWHM of 7.0 mm was for Q.Clear with *β* of 100. A FWHM of 9 mm was measured for FBP with 5 mm filter. Relative to this, the largest difference was measured for FBP with a 15-mm filter (− 7.5 mm), while the smallest was for Q.Clear with β400 (0.0 mm).

The Q.Clear reconstruction with *β* of 100 yielded the poorest uniformity of 15.8%, while the highest uniformity was for FBP with a filter of 15 mm (8.8%) (Fig. 6). Relative to the FBP with 5 mm filter, the largest difference (− 3.9) was for Q.Clear with *β* of 100; while the smallest difference (− 0.03) was for Q.Clear with *β* of 700.

For SNR, the highest value (65.3) was for Q.Clear with *β* of 1000 (Fig. 7). The poorest SNR was for FBP with 5 mm filter (19.3). Relative to this, the largest difference (− 45.9) was for Q.Clear with *β* of 1000, while the smallest difference (− 6.6) was for FBP with 10 mm filter.

## Discussion

This study investigated the performance of the Q.Clear reconstruction algorithm using a PET-MR against OSEM (PET-MR and PET-CT) and FBP (PET-CT) algorithms on general use and brain phantom data. Different isotopes were used to characterise noise, uniformity, SNR and quantitative bias outcomes, and the Hoffman brain phantom was also selected to simulate radioisotope distribution in the grey and white matter of the brain.

Carbon-11 and Fluorine-18 tracers are used in clinical and research PET not only because of their short half-life but also due to the short-range of the positrons in tissue [27]. Our study demonstrates that the results obtained for the spatial resolution, signal-to-noise and axial uniformity metrics, present very similar patterns when using the Hoffman phantom filled with ^18^F or ^11^C. This data is in accordance with Conti et al.’s [27] findings using the NEMA phantom filled with pure β^+^ emitters and scanned up until 200 million net true counts were obtained. In their study, the ^18^F and ^11^C images presented very similar radial profiles.

Our NEMA phantom data demonstrate that as the Q.Clear *β* value increases, the contrast recovery and background variability decrease. Using the same phantom filled with ^18^F-FDG and a GE Discovery 690 PET/CT scanner, Teoh et al. also found that when Q.Clear *β* values increased, the contrast recovery and background variability decreased [28]. Furthermore, our data shows that the contrast recovery results obtained are lower for the FBP and OSEM reconstructions (performed on the PET-CT and on the PET-MR) than for the Q.Clear reconstructions. This is also in line with Teoh et al.’s findings, as the group reported the lowest contrast recovery results when using the OSEM reconstruction versus Q.Clear reconstructions. As expected, our data shows that as the sphere diameter increases from 10 to 17 mm (hot spheres) and from 30 to 39 mm (cold spheres), the contrast recovery also increases, in line with previous work [28].

The background variability results are higher for Q.Clear than for OSEM when reconstructing data on the PET-MR. This is in contrast with Teoh et al.’s findings in the PET-CT scanner as in the study mentioned above the group reported OSEM background variability results higher or equal to the background variability results obtained with Q.Clear with *β* > 200 [28]. This may be partly due to the differences in the width of filter used (2 mm and 6.4 mm in Teoh et al.’s study vs 5 mm, 10 mm and 15 mm used in our study) and to the use of Point Spread function modelling in Teoh et al.’s study [28]. The FBP and OSEM background results on the PET-CT are very similar. Interestingly, unlike the OSEM background variability results obtained in the PET-CT which present a slight upwards trend, the OSEM PET-MR results present a downwards trend, as the sphere diameter increases. This downwards trend is consistent with the findings from Caribé et al., who scanned an ^18^F-filled phantom in the GE Signa PET-MR and reconstructed the acquired dataset with TOF-OSEM with 4 iterations and 28 subsets. The team obtained a background variability of 6.1% for the sphere with 10 mm decreasing with the increase in sphere diameter to 2.7% for the 37 mm sphere [17]. Reynés-Llompart et al. scanned a ^18^F-filled NEMA phantom on a GE Discovery IQ PET-CT scanner. They found that as *β* values increased, the background variability and the contrast recovery coefficients decreased [29].

The FWHM(*x*,*y*) and FWHM(*z*) results show that the Q.Clear reconstructions with different *β* values on the PET-MR are more closely related to the FBP reconstruction, with a 5-mm kernel, rather than the FBP reconstructions with the 10 mm and 15 mm kernel in the PET-CT. The FWHM(*x*,*y*) results obtained for the Q.Clear reconstructions in the PET-MR are lower although still related to the results obtained for the FBP reconstruction with 5 mm filter in the PET-CT. The FWHM(z) results obtained for the Q.Clear reconstructions with *β* < 400 are considerably lower than the ones obtained for the FBP and OSEM reconstructions performed in the PET-CT. These metrics indicate an improvement in the in plane and axial resolution with this algorithm. This is consistent with the data obtained by Rogasch et al., who scanned a NEMA phantom during 30 min in a GE Discover MI PET-CT system and reconstructed the data with TOF-OSEM 4 iterations, 16 subsets and 2 mm filter, TOF-OSEM 2 iterations, 17 subsets and 2 mm filter, TOF-OSEM 2 iterarions, 8 subsets and 6.4 mm filter, Q.Clear β150, Q.Clear β300 and Q.Clear β450 [20]. The group reconstructed the spatial resolution from the radial activity profiles of the 37 mm sphere and found that all the Q.Clear reconstructions resulted in better spatial resolution results than TOF-OSEM [20].

Uniformity is strongly dependent on the *β* value and for *β* < 600, it can be worse than the uniformity obtained with the FBP reconstruction. Additionally, as the *β* value increases, so does the signal-to-noise and the difference to the FBP reconstructions. This data matches the visual image quality and is consistent with reports from clinical scans and other studies [30–34]. The uniformity and SNR results are explained by the fact that the *β* value acts as a noise suppression term and penalizes the differences in image intensity between bordering pixels [34].

Overall, Q.Clear with lower *β* levels improves FWHM(*x*,*y*) and FWHM(*z*), whereas Q.Clear with higher *β* levels improves uniformity and SNR. The findings in our study which was conducted in a GE Signa PET-MR scanner are consistent with those obtained by Reynés-Llompart et al. on a GE Discovery IQ PET-CT scanner. The team conducted a clinical evaluation of torso and brain acquisition and found that, after subjective quality assessment, *β* values between 300 and 400 are recommended for reconstructing torso acquisitions and *β* values between 100 and 200 are recommended for brain acquisitions [29].

## Conclusion

Q.Clear improves contrast recovery on the PET-MR in comparison to OSEM. Moreover, Q.Clear also provides better in plane, axial resolution and signal-to-noise; however, its effect on image uniformity requires further investigations. For brain PET studies, in which spatial resolution is paramount, the Q.Clear reconstruction with *β* value of 100 will provide the best results based on our novel data with the Hoffman phantom, albeit with lower SNR compared with *β* value of 1000 and equivalent values to FBP.

## Supplementary Information

**Additional file 1: Supplementary file 1.** Fig. 1 Bland-Altman plots assessing agreement between FBP with 5mm and Q.Clear with different β values, for the contrast recovery data obtained from the NEMA phantom. FBP 5mm and Q.Clear β100 (A); FBP 5mm and Q.Clear β200 (B); FBP 5mm and Q.Clear β300 (C); FBP 5mm and Q.Clear β400 (D); FBP 5mm and Q.Clear β500 (E). Fig. 2 Bland-Altman plots assessing agreement between FBP with 5mm and Q.Clear with different β values, for the contrast recovery data obtained from the NEMA phantom. FBP 5mm and Q.Clear β600 (F); FBP 5mm and Q.Clear β700 (G); FBP 5mm and Q.Clear β800 (H); FBP 5mm and Q.Clear β900 (I); FBP 5mm and Q.Clear β1000 (J).

**Additional file 2: Supplementary file 2.** Fig. 1 Bland-Altman plots assessing agreement between TOF-OSEM 4iteration, 8subsets with 5mm (4i8s5mm) and Q.Clear with different β values, for the contrast recovery data obtained from the NEMA phantom. TOF-OSEM 4i8s5mm and Q.Clear β100 (A); TOF-OSEM 4i8s5mm and Q.Clear β200 (B); TOF-OSEM 4i8s5mm and Q.Clear β300 (C); TOF-OSEM 4i8s5mm and Q.Clear β400 (D); TOF-OSEM 4i8s5mm and Q.Clear β500 (E). Fig. 2 Bland-Altman plots assessing agreement between TOF-OSEM 4iteration, 8subsets with 5mm (4i8s5mm) and Q.Clear with different β values, for the contrast recovery data obtained from the NEMA phantom. TOF-OSEM 4i8s5mm and Q.Clear β600 (F); TOF-OSEM 4i8s5mm and Q.Clear β700 (G); TOF-OSEM 4i8s5mm and Q.Clear β800 (H); TOF-OSEM 4i8s5mm and Q.Clear β900 (I); TOF-OSEM 4i8s5mm and Q.Clear β1000 (J).

**Additional file 3: Supplementary file 3.** Fig. 1 Bland-Altman plots assessing agreement between FBP with 5mm and Q.Clear with different β values, for the background variability data obtained from the NEMA phantom. FBP 5mm and Q.Clear β100 (A); FBP 5mm and Q.Clear β200 (B); FBP 5mm and Q.Clear β300 (C); FBP 5mm and Q.Clear β400 (D); FBP 5mm and Q.Clear β500 (E). Fig. 2 Bland-Altman plots assessing agreement between FBP with 5mm and Q.Clear with different β values, for the background variability data obtained from the NEMA phantom. FBP 5mm and Q.Clear β600 (F); FBP 5mm and Q.Clear β700 (G); FBP 5mm and Q.Clear β800 (H); FBP 5mm and Q.Clear β900 (I); FBP 5mm and Q.Clear β1000 (J).

**Additional file 4: Supplementary file 4.** Fig. 1 Bland-Altman plots assessing agreement between TOF-OSEM 4iteration, 8subsets with 5mm (4i8s5mm) and Q.Clear with different β values, for the background variability data obtained from the NEMA phantom. TOF-OSEM 4i8s5mm and Q.Clear β100 (A); TOF-OSEM 4i8s5mm and Q.Clear β200 (B); TOF-OSEM 4i8s5mm and Q.Clear β300 (C); TOF-OSEM 4i8s5mm and Q.Clear β400 (D); TOF-OSEM 4i8s5mm and Q.Clear β500 (E). Fig. 2 Bland-Altman plots assessing agreement between TOF-OSEM 4iteration, 8subsets with 5mm (4i8s5mm)and Q.Clear with different β values, for the background variability data obtained from the NEMA phantom. TOF-OSEM 4i8s5mm and Q.Clear β600 (F); TOF-OSEM 4i8s5mm and Q.Clear β700 (G); TOF-OSEM 4i8s5mm and Q.Clear β800 (H); TOF-OSEM 4i8s5mm and Q.Clear β900 (I); TOF-OSEM 4i8s5mm and Q.Clear β1000 (J).

## Data Availability

The datasets generated and analysed during the current study are not publicly available due to proprietary restrictions but are available from the corresponding author on reasonable request.

## Abbreviations

PET: Positron emission tomography
FBP: Filtered back-projection
OSEM: Ordered subset expectation maximization
BSREM: Block sequential regularized expectation maximization
BPL: Bayesian penalized likelihood
EM: Expectation-maximization
GE: General Electric
CT: Computed tomography
MR: Magnetic resonance
NEMA: National Electrical Manufacturers Association
IQ: Image quality
kBq: Kilobecquerel
mL: Millilitre
TOF: Time of flight
3D: Three-dimensional
IDL: Interactive Data Language
ROI: Regions of interest
FWHM: Full-width half maximum
VOI: Volume of interest
SNR: Signal-to-noise ratio
cc: Cubic centimetre
